# Clinical and genetic analysis of 2 rare cases of Wiskott–Aldrich syndrome from Chinese minorities: Two case reports

**DOI:** 10.1097/MD.0000000000025527

**Published:** 2021-04-23

**Authors:** Haifeng Liu, Yanchun Wang, Yangfang Li, Lvyan Tao, Yu Zhang, Xiaoli He, Yuantao Zhou, Xiaoning Liu, Yan Wang, Li Li

**Affiliations:** a Kunming Key Laboratory of Children Infection and Immunity, Yunnan Key Laboratory of Children's Major Disease Research, Yunnan Medical Center for Pediatric Diseases, Yunnan Institute of Pediatrics; b Department of 2nd Infections; c Department of Neonatology; d Department of Pharmacy, Kunming Children's Hospital, Kunming, Yunnan, China.

**Keywords:** next-generation sequencing, thrombocytopenia, tuberculosis, *WAS* gene, Wiskott–Aldrich syndrome

## Abstract

**Rationale:**

Wiskott–Aldrich syndrome (WAS) is a rare X-linked recessive disease characterized by thrombocytopenia, small platelets, eczema, immunodeficiency, and an increased risk of autoimmunity and malignancies. X-linked thrombocytopenia (XLT), the milder phenotype of WAS, is always limited to thrombocytopenia with absent or slight infections and eczema. Here, we illustrated the clinical and molecular characteristics of 2 unrelated patients with WAS from Chinese minorities.

**Patient concerns:**

Patient 1, a 13-day-old male newborn of the Chinese Lahu minority, showed a classic WAS phenotype, including thrombocytopenia, small platelets, buttock eczema, and recurrent infections. Patient 2, an 8-year-and 8-month-old boy of the Chinese Zhuang minority, presented an XLT phenotype without eczema and repeated infections.

**Diagnosis:**

Next-generation sequencing was performed to investigate the genetic variations. Flow cytometry was used to quantify the expression of WAS protein and analyze the lymphocyte subsets. A novel frameshift *WAS* mutation (c.927delC, p.Q310Rfs∗135) and a known nonsense *WAS* mutation (c.1090C>T, p.R364X) were identified in Patient 1 and Patient 2, respectively. Both patients were confirmed to have WAS protein deficiency, which was more severe in Patient 1. Meanwhile, the analysis of lymphocyte subsets revealed an abnormality in Patient 1, but not in Patient 2. Combined with the above clinical data and genetic characteristics, Patient 1 and Patient 2 were diagnosed as classic WAS and XLT, respectively. In addition, many miliary nodules were accidentally found in abdominal cavity of Patient 2 during appendectomy. Subsequently, Patient 2 was confirmed with pulmonary and abdominal tuberculosis through further laboratory and imaging examinations. To our knowledge, there have been only a few reports about WAS/XLT with tuberculosis.

**Interventions:**

Both patients received anti-infection therapy, platelet transfusions, and intravenous immunoglobulins. Moreover, Patient 2 also received antituberculosis treatment with ethambutol and amoxicillin-clavulanate.

**Outcomes:**

The clinical symptoms and hematological parameters of these 2 patients were significantly improved. Regrettably, both patients discontinued the treatment for financial reasons.

**Lessons:**

Our report expands the pathogenic mutation spectrum of *WAS* gene and emphasizes the importance of molecular genetic testing in diagnosing WAS. Furthermore, researching and reporting rare cases of WAS from different populations will facilitate diagnosis and treatment of this disease.

## 1 Introduction

Wiskott–Aldrich syndrome (WAS, OMIM #301000) is a rare and severe X-linked recessive primary immunodeficiency disease caused by *WAS* gene mutations, with an approximate incidence of 1/1,000,000 to 10/1,000,000.^[1–5]^ Due to different *WAS* gene mutations, there are diverse clinical phenotypes, ranging from classic WAS to X-linked thrombocytopenia (XLT, OMIM #313900) and X-linked neutropenia (OMIM #300299). Classic WAS has characteristic clinical manifestations, including thrombocytopenia, small platelet, eczema, immunodeficiency, and an increased risk of autoimmune diseases and malignancies.^[1–3]^ Persistent thrombocytopenia (<70.00 × 10^9^/L) with small platelets is the most significant hematological characteristic of WAS. About 86% of patients with WAS develop the initial symptoms mainly characterized by petechiae or ecchymosis 1 month after birth.^[6]^ In addition to mild symptoms such as petechiae and ecchymosis, severe thrombocytopenia may also result in fatal intracranial and gastrointestinal bleeding. What's more, eczema is observed in over 80% of WAS patients, with the coverage and severity varying greatly.^[3]^ The development of eczema is associated with Th1/Th2 imbalance caused by functional abnormalities of Treg cells.^[7,8]^ Recurrent infection due to immunodeficiency is also a common clinical manifestation of WAS and severe infection is one of the leading causes of the ultimate death in WAS patients.^[9]^ Furthermore, WAS patients are also susceptible to autoimmunity and malignancies. The previous data show that 40% to 72% of patients with WAS developed autoimmune disorders due to disrupted immune tolerance, among which autoimmune hemolytic anemia (36%) and arthritis (29%) were more frequently observed.^[3,10]^ Malignancies (80% lymphomas) are present in 10% to 15% of classic WAS patients with an average age of onset of 9.5 years.^[11–13]^ Notably, WAS patients with milder phenotype are clinically classified as XLT, which is mainly characterized by low platelet count and small platelets with or without mild infections and eczema,^[14]^ and X-linked neutropenia is characterized by neutropenia and myelodysplasia.^[15]^

**Figure 1:**
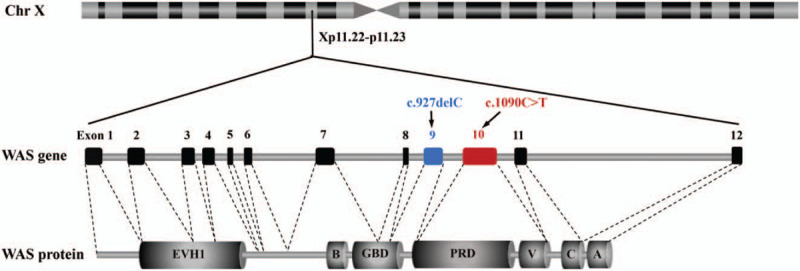
Schematic diagram of *WAS* gene structure, location, and corresponding encoded Wiskott–Aldrich syndrome protein (WASp). *WAS* gene is located at X chromosome p11.22-p11.23 and contains 12 exons. The *WAS* c.927delC mutation (in Patient 1) and *WAS* c.1090C>T mutation (in Patient 2) occurred on exons 9 (highlighted in blue) and 10 (highlighted in red), respectively. WASp contains 5 functional domains, namely Ena-VASP homology 1 domain (EVH1), basic domain (B), guanosine triphosphatase-binding domain (GBD), proline-rich domain (PRD), and verprolin homology (V)/central hydrophobic region (C)/acidic region (A) domain (VCA). The corresponding regions of WASp encoded by exon 1 to 12 are pointed out by the black dotted line.

**Figure 2:**
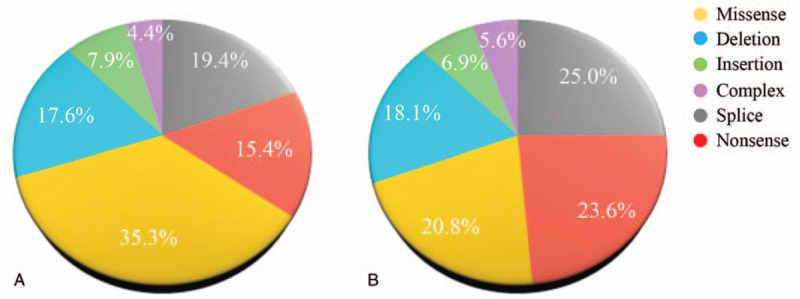
Statistics diagram of *WAS* mutation distribution for patients with WAS. (A) Distribution of *WAS* mutation types in European-American patients with WAS. (B) Distribution of *WAS* mutation types in Chinese patients with WAS. WAS = Wiskott–Aldrich syndrome.

*WAS* gene was identified by Derry et al through positional cloning in 1994.^[16]^ The gene is located at the short arm of the X chromosome (Xp11.22-p11.23), contains 12 exons and encodes a WAS protein (WASp) composed of 502 amino acids (Fig. 1). WASp is a member of the actin nucleation-promoting factor family, widely found in the cytoplasm of hematopoietic cells, and involved in cellular signal transduction and cytoskeletal remodeling.^[17,18]^ WASp contains 5 functional domains (Fig. 1), namely Ena-VASP homology 1 domain, a short basic domain (B), guanosine triphosphatase-binding domain (GBD), proline-rich domain (PRD), and verprolin homology/central hydrophobic region/acidic region (VCA) domain from the N-terminus to the C-terminus. When not activated, WASp is in an auto-inhibited conformation, where GBD and VCA domains interact to form a hairpin-like closed structure that inhibits the actin polymerization and the actin-associated protein (actin-related proteinS 2/3 [Arp2/3]) complex activation; WASp can be activated by a variety of signals, including the binding of cell division cycle 42-Guanosine triphosphate to the GBD, the binding of adaptor protein, such as noncatalytic region of tyrosine kinase to PRD, and phosphorylation of Y291, thereby relieving the auto-inhibited state, regulating the polymerization of downstream actin and the remodeling of cytoskeleton, and playing an important role in the formation of immune synapses and the exertion of hematopoietic cell function.^[19–21]^ Since the identification of *WAS* gene in 1994, more than 400 different mutations have been reported.^[9]^ Previous researches have shown that 35.3% of *WAS* gene mutations in European and American patients are missense mutations, and the rest includes splicing mutations (19.4%), deletion mutations (17.6%), nonsense mutations (15.4%), insertion mutations (7.9%), and complex mutations (4.4%) (Fig. 2A),^[22]^ while the distribution of the above mutation types in Chinese WAS patients is 20.8%, 25.0%, 18.1%, 23.6%, 6.9%, and 5.6%, respectively (Fig. 2B).^[6]^ Deletion or low expression of WASp due to pathogenic mutations in *WAS* gene will destroy the function of immune cells to varying degrees, lead to combined immunodeficiency and the imbalance between immune tolerance and autoimmunity, and eventually manifest with severe infections, autoimmune diseases, and malignancies.^[23–27]^ Herein, we reported 2 rare cases of WAS in Chinese ethnic minorities, investigated the clinical and molecular characteristics of these 2 unrelated patients with WAS, and described their respective treatments.

## 2 Materials and methods

### 2.1 Subjects and clinical evaluations

Patient 1 was a 13-day-old male newborn of Lahu minority and Patient 2 was an 8-year-and 8-month-old boy of the Zhuang minority. There was no consanguineous marriage in their respective families. Both patients and their parents were included in this study after the parents had signed the written informed consent. We performed detailed clinical assessments on the patients, including physical examination, associated laboratory test, and routine imaging examination. This study was approved by the Ethics Committee of Kunming Children's Hospital and strictly followed the Declaration of Helsinki.

### 2.2 Next-generation sequencing (NGS) and data analysis

All genetic tests were conducted in accordance with relevant Chinese laws. Genomic DNA was extracted from the peripheral blood of these 2 patients by QIAamp DNA Mini Kit (Qiagen, Shanghai, China). Nanodrop 2000 (Thermo Fisher Scientific, DE), an ultramicro spectrophotometer, was used to evaluate and quantify the extracted DNA. The qualified genomic DNA was fragmented into 350 to 450 bp and end-tagged with an adaptor sequence.

Next, the whole exon region was selected by the GenCap custom enrichment kit (MyGenostics Inc., Beijing, China) following the manufacturer's instructions. In detail, the biotinylated probe (80–120-mer) was hybridized with prepared library DNA, and the streptavidin-modified magnetic beads were covalently bound to the biotinylated probe (MyGenostics) to capture the targeted gene. The magnetic beads carrying the target gene were adsorbed by the magnetic frame. Subsequently, the captured DNA was obtained by washing the adsorbed magnetic beads with WB1 buffer (25°C, 15 minutes) and WB3 buffer (65°C, 10 minutes), respectively. The obtained DNA was purified with 80% ethanol and eluted with elution buffer (30 minutes). Finally, NGS was performed on NextSeq500 (Illumina, San Diego, CA).

Following sequencing, bioinformatics analysis of the raw data was performed. Low-quality/adapter reads were removed by Cutadapt 1.16. The preprocessed data were compared with the human genome (hg19) by Burrows-Wheeler Aligner software 0.7.10. The deletion of redundant reads generated by polymerase chain reaction (PCR) amplification, the recalibration of the base quality score of remaining reads, as well as the detection of single nucleotide polymorphisms and deletions and insertions were performed with Genome Analysis Toolkit software (GATK 4.0.8.1). Annovar software was used to annotate the identified single nucleotide polymorphisms and deletions and insertions. Then, the suspected causative mutations were screened and the pathogenicity of identified mutation sites was assessed in strict accordance with the guidelines of the American College of Medical Genetics and Genomics.

### 2.3 Sanger sequencing

Genomic DNA was extracted from peripheral blood of other family members (respective parents of Patient 1 and Patient 2) for Sanger sequencing to verify the results of NGS. According to the likely pathogenic mutations obtained by NGS, primers were designed using PRIMER 5 software for PCR amplification. After purification, the PCR products were sequenced on ABI PRISM 3730 genetic analyzer (Applied Biosystems; Thermo Fisher Scientific, Inc.) and the obtained sequence data were compared with human reference sequence of *WAS* gene.

### 2.4 Flow cytometry analysis

#### 2.4.1 Expression levels of WASp

Flow cytometry was used to examine the expression of WASp in peripheral blood mononuclear cells (PBMCs). PBMCs were isolated from peripheral blood of both patients and their parents by human PBMCs separation kit (Solarbio, Beijing, China), washed twice with phosphate-buffered saline (PBS), and centrifuged at 1650 rpm for 10 minutes. Cell fixation (10 minutes) and permeabilization (15 minutes) were performed successively using the perfix-nc Kit (Beckman Coulter, A07803) following the manufacturer's instructions. Next, 1:150 diluted Rabbit anti-WASp (Abcam, ab75830, UK) was added and the samples were incubated at room temperature for 30 minutes. After centrifugation at 2000 rpm for 5 minutes at 4°C, the supernatant was discarded, while the precipitated cells were washed again and resuspended in 1 mL PBS (precooling at 4°C). Finally, 1:200 diluted Goat anti-Rabbit IgG H&L (Fluorescein isothiocyanate; Abcam, ab205718) was added for staining in the dark. Flow cytometry was performed with the ACEA NovoCyte flow cytometer (ACEA Biosciences, San Diego, CA) and the data were analyzed by FlowJo v10 software.

#### 2.4.2 Lymphocyte subsets

Peripheral blood lymphocytes of both patients were isolated using lymphocyte separation medium (TBD Science, Tianjin, China) and washed twice with 3 mL PBS. Then, cells were resuspended in 1 mL precooled PBS, followed by staining with the BD Multitest^TM^ 6-Color kit (BD Biosciences) and incubating for 30 minutes under light-protected conditions at room temperature. The stained lymphocytes were analyzed on ACEA NovoCyte flow cytometer (ACEA Biosciences) and FlowJo v10 software.

## 3 Results

### 3.1 Clinical characteristics

#### 3.1.1 Patient 1

Patient 1 was a 13-day-old male newborn of Chinese Lahu minority with full-term normal delivery. His mother had a history of 10 pregnancies: 2 older brothers of the patient died at an early age due to unknown causes, 2 older sisters were healthy individuals, and the remaining parities were aborted spontaneously. Based on the guardians’ will, the patient's sisters were not recruited in the study. The patient received clinical evaluations and treatments in the local county hospital due to skin petechiae on the second day after birth. He was diagnosed with neonatal infection and neonatal hemorrhage (laboratory tests in the hospital showed that the platelet count was decreased to 33.00 × 10^9^/L) and received anti-infective (cefoperazone-sulbactam and penicillin) and hemostatic therapies (vitamin K1, aminomethylbenzoic acid, and etamsylate). The infection improved after 9 days, but no significant regression of the skin petechiae was observed. He was subsequently transferred to the Kunming Children's Hospital for further examinations and treatments.

**Figure 3:**
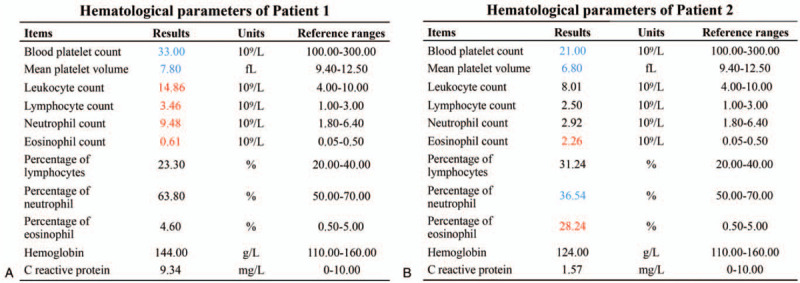
The results of hematological examinations. (A) Hematological parameters of Patient 1. Compared to the reference range, the platelet count and mean platelet volume (MPV) were significantly decreased, the leukocyte count, lymphocyte count, neutrophil count, and eosinophil count were increased to varying degrees, and other parameters were normal. (B) Hematological parameters of Patient 2. Compared to the reference range, the platelet count, MPV, and neutrophil percentage were decreased, the eosinophil count and eosinophil percentage were increased, and no other abnormalities were found. Parameters above the reference range are marked in red, and parameters below the reference range are marked in blue.

Physical examination after admission revealed that the patient had significant petechiae on the heel and eczema on the buttocks. The results of hematological examination showed that the platelet count (33.00 × 10^9^/L) and mean platelet volume (MPV) (7.80 fL) were significantly decreased, the leukocyte count (14.86 × 10^9^/L), lymphocyte count (3.46 × 10^9^/L), neutrophil count (9.48 × 10^9^/L), and eosinophil count (0.61 × 10^9^/L) were increased to varying degrees, and the rest of the parameters were normal (Fig. 3A).

**Figure 4:**
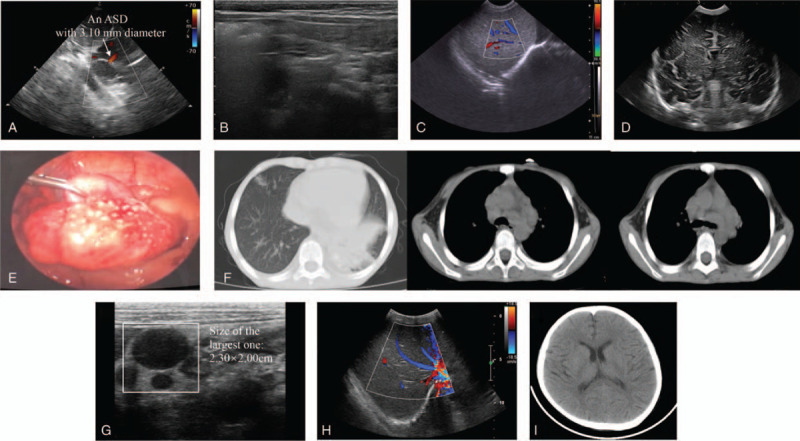
Imaging findings of these 2 patients. (A) Ultrasonic cardiography of Patient 1. An atrial septal defect (ASD) with a diameter of 3.10 mm was observed. The ASD was indicated by the white arrow. (B) Abdominal color ultrasound of Patient 1. No abnormalities were observed. (C) Color ultrasound of abdominal organs from Patient 1. No abnormalities were found. (D) Brian color Doppler ultrasound of Patient 1. No pathological signs were shown. (E) Laparoscopic findings during the appendectomy of Patient 2. Many miliary nodules distributed in the abdominal cavity were observed. (F) Chest computed tomography (CT) of Patient 2. Bilateral lungs showed inflammatory lesions, the lower lobe of the left lung showed patchy high-density lesions, and lymphadenopathy was observed in the mediastinum and hilar regions. (G) Abdominal color Doppler ultrasound of Patient 2. Multiple mesenteric lymph nodes were detected in the right lower abdomen and around the umbilicus, with the largest one of about 2.30 × 2.00 cm. Lymph nodes were indicated by the white box. (H) Color Doppler ultrasound of abdominal organs from Patient 2. There were no apparent abnormalities in liver, gallbladder, pancreas, spleen, and both kidneys. (I) Brain CT scan of Patient 2. No abnormal density shadow was found in brain parenchyma.

In addition, a grade II/VI systolic murmur was noted during auscultation in the valvular area of the heart, which was consistent with the ultrasonic cardiography (UCG) results that an atrial septal defect (ASD) (diameter 3.10 mm) was observed in the patient's heart and blood flow was shunted from left atrium to right atrium at the site of the defect (Fig. 4A). No pathological signs were found in color Doppler ultrasound of the abdominal cavity (Fig. 4B), abdominal organs (liver, gallbladder, pancreas, spleen, and both kidneys) (Fig. 4C), and brain (Fig. 4D).

#### 3.1.2 Patient 2

Patient 2 was an 8-year-and 8-month-old boy of the Chinese Zhuang minority. He was admitted to Kunming Children's Hospital with scattered subcutaneous bleeding spots on both lower limbs. No eczema was found through physical examination. The hematological examination was performed, and the results showed that the platelet count (21.00 × 10^9^/L), MPV (6.80 fL), and neutrophil percentage (36.54%) were decreased, the eosinophil count (2.26 × 10^9^/L) and eosinophil percentage (28.24%) were increased, and no obvious abnormalities were observed in other hematological parameters (Fig. 3B). According to medical history, the patient had also experienced asymptomatic thrombocytopenia (10.00 × 10^9^/L) at the age of 1 year, which was resolved spontaneously without any treatment.

In addition, the patient underwent appendectomy for acute suppurative appendicitis, during which time many miliary nodules distributed in the abdominal cavity were accidentally found by laparoscope (Fig. 4E). The relevant medical history and family history revealed that the patient had recurrent low-grade fever for the past 6 months and his father was a patient with active tuberculosis. Detailed examinations for tuberculosis were performed after surgery. Both results of the interferon gamma release assay and the tuberculin purified protein derivative test were positive, and the chest computed tomography scan showed inflammatory lesions in bilateral lungs, prominent patchy hyperdense lesions in the lower lobe of the left lung, and hilar and mediastinal lymphadenopathy (Fig. 4F). Abdominal color Doppler ultrasound showed multiple mesenteric lymph nodes in the right lower abdomen and around the umbilicus, with the largest one of approximately 2.30 × 2.00 cm (Fig. 4G). Both color Doppler ultrasound of abdominal organs (liver, gallbladder, pancreas, spleen, and kidney) (Fig. 4H) and brain computed tomography revealed no apparent abnormalities (Fig. 4I).

### 3.2 Identification of *WAS* mutations

#### 3.2.1 A novel frameshift mutation (c.927delC) of *WAS* gene was identified in Patient 1

**Figure 5:**
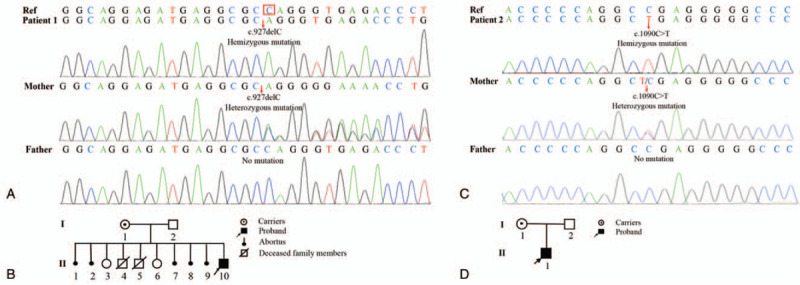
Results of mutation analysis. (A) Identification of a novel *WAS* gene mutation in Patient 1 and his mother. A single nucleotide “C” deletion (c.927delC) was found in the *WAS* gene of Patient 1. This heterozygous mutation was also detected in his mother, while no mutation was detected in his father. The red arrow and box indicate the deletion locus. (B) Pedigree of Patient 1's family. Patient 1's mother (I-1) was only a carrier of the *WAS* c.927delC mutation without any clinical symptoms, while his father (I-2) had no mutation. Patient 1 (II-10) was identified with a hemizygous mutation and presented with a classic WAS phenotype. Both of his older brothers (II-4, 5) died due to unknown causes and both of his older sisters (II-3, 6) were healthy, while II-1, 2, 7, 8, 9 were aborted spontaneously. (C) A known nonsense mutation of *WAS* gene was detected in Patient 2 and his mother. “C” was replaced by “T” at position c.1090 (c.1090C>T) of the *WAS* gene, and the codon CGA encoding arginine was changed to the termination codon TGA. This mutation was not found in his father. The red arrow indicates where the mutation occurred. (D) Pedigree of Patient 2's family. The proband (Patient 2, II-1) was found to be hemizygous for the mutation c.1090C>T in *WAS* gene, his mother (I-1) had this heterozygous mutation at the same locus with no symptoms of WAS, and no mutations were found in his father (I-2). WAS = Wiskott–Aldrich syndrome.

The NGS were used to assess the patients and their parents at the genetic level. The results showed that Patient 1 carried a novel hemizygous mutation in *WAS* gene. Specifically, there was a single nucleotide “C” deletion at the site of c.927 (c.927delC) in exon 9 of the *WAS* gene. The deletion resulted in a frameshift mutation causing changes in 135 amino acids from p.310 and induced a premature stop codon (p.Q310Rfs∗135) (Fig. 5A). The stop codon induced by c.927delC resulted in a large truncation of WASp. No report on this mutation (c.927delC, p.Q310Rfs∗135) in *WAS* gene has been found in the Human Gene Mutation Database. This mutation was likely to be the pathogenic mutation contributing to the clinical phenotype of Patient 1. Sanger sequencing was performed to validate the mutation. The sequencing results of his parents indicated that his mother also carried c.927delC heterozygous variant, while no mutations were detected in his father (Fig. 5A). The *WAS* c.927delC mutation that occurred in Patient 1 was inherited from his mother, which was consistent with inheritance mode of WAS (X-linked recessive inheritance). Based on the above results, we speculated that Patient 1 suffered from WAS and his mother was only an asymptomatic carrier of the *WAS* c.927delC mutation. Unfortunately, both of his older brothers died at an early age due to unknown etiology (Fig. 5B), and it was presumed that they also suffered from WAS.

#### 3.2.2 A known nonsense mutation of *WAS* gene (c.1090C>T) was detected in Patient 2

Patient 2 was found to carry a known nonsense mutation, which was confirmed to be a single-nucleotide substitution from “C” to “T” at position c.1090 (c.1090C>T) in exon 10 of the *WAS* gene (Fig. 5C). This mutation resulted in an amino acid change p.R364X. The Sanger sequencing of his parents indicated that his mother had the heterozygous c.1090C>T mutation in *WAS* gene, but no mutations were detected in his father. Meanwhile, his mother is only a carrier of the mutation with no associated symptoms of WAS (Fig. 5D). This pathogenic mutation has been previously reported by Lemahieu et al,^[28]^ and is closely related to the pathogenesis of WAS milder phenotype- XLT, which coincides with the clinical phenotype of Patient 2.

### 3.3 Various degrees of WASp deficiency were detected in Patient 1 and Patient 2

**Figure 6:**
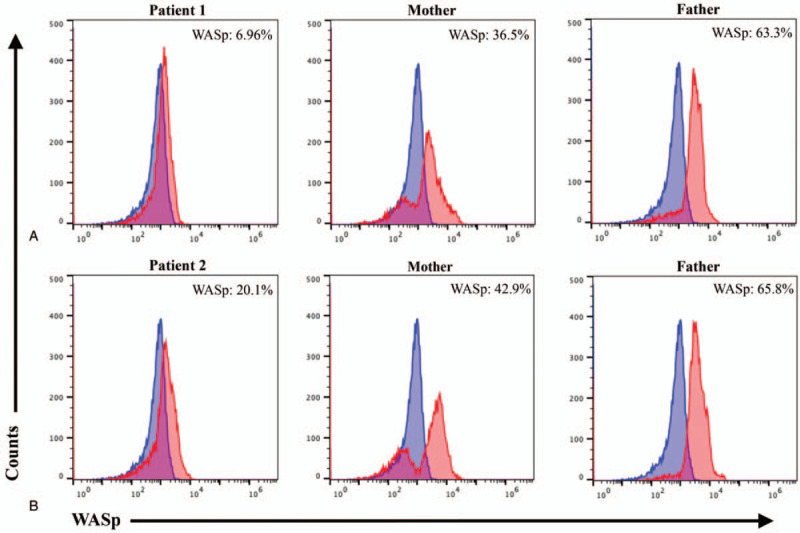
Flow cytometry was used to assess the expression levels of Wiskott–Aldrich syndrome protein (WASp). (A) WASp expression levels in peripheral blood mononuclear cells (PBMCs) from Patient 1 and his parents. A single peak (red), which almost completely overlapped with the blank control peak (dark blue), was detected in Patient 1. The expression of WASp was 6.96%. Double peaks (red) were detected in his mother, one of which partially overlapped with the blank control peak (dark blue) and the other was shifted. The expression of WASp was 36.5%. A single shifted peak (red) was identified in his father, and the expression of WASp was 63.3%. (B) WASp expression levels in PBMCs from Patient 2 and his parents. Patient 2 and his father both had single peaks (red), and the WASp expressions in them were 20.1% and 65.8%, respectively. In his mother, double peaks (red), including a partially overlapping peak and a shifted peak, were detected. The expression of WASp was 42.9%. The peaks of all subjects are marked in red, while the blank control peak is marked in dark blue.

In order to further verify the pathogenicity of the 2 mutations identified by genetic testing, the fluorescence intensity was measured by flow cytometry to quantitatively analyze the expression levels of WASp in PBMCs isolated from all subjects. The WASp expression of Patient 1 was 6.96%, which was significantly lower than that of his mother (36.5%) and father (63.3%) (Fig. 6A). While the expression of WASp was relatively mildly downregulated in Patient 2 (20.1%), and the WASp expressions in his mother and father were 42.9% and 65.8%, respectively (Fig. 6B). According to the above results, both patients had low WASp expression in PBMCs, and the deficiency of WASp in Patient 1 was more severe than that in Patient 2.

### 3.4 Analytical results of lymphocyte subsets

**Figure 7:**
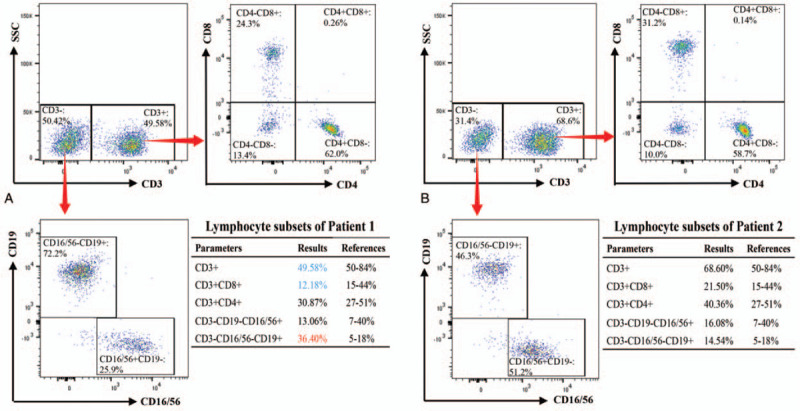
Flow cytometry analysis of lymphocyte subsets. (A) Analysis results of lymphocyte subsets from Patient 1. The percentages of total T lymphocytes (CD3+: 49.58%) and CD8+ T lymphocytes (CD3+CD8+: 12.18%) were decreased, the proportion of B cells (CD3-CD16/56-CD19+: 36.40%) was increased, and other parameters were normal. (B) Analysis results of lymphocyte subsets from Patient 2. No obvious abnormalities were found. Red arrows in the scatter plots indicate the analysis routes. In the diagrams of analysis results for lymphocyte subsets, parameters above the reference range are indicated in red and parameters below the reference range are indicated in blue.

Lymphocyte subsets were evaluated by flow cytometry to analyze the immune status of these 2 patients. For Patient 1, the percentages of total T lymphocytes (CD3+: 49.58%) and CD8+ T lymphocytes (CD3+CD8+: 12.18%) were decreased, the proportion of B cells (CD3-CD16/56-CD19+: 36.40%) was increased, and other parameters were normal (Fig. 7A). However, the analysis of lymphocyte subsets in Patient 2 revealed no significant abnormalities (Fig. 7B). In addition, although CD4+ T lymphocytes were at normal levels in both patients, the proportion of CD4+ T lymphocytes in Patient 1 (CD3+CD4+: 30.87%) was lower than that in Patient 2 (CD3+CD4+: 40.36%).

### 3.5 Treatments and outcomes

**Figure 8:**
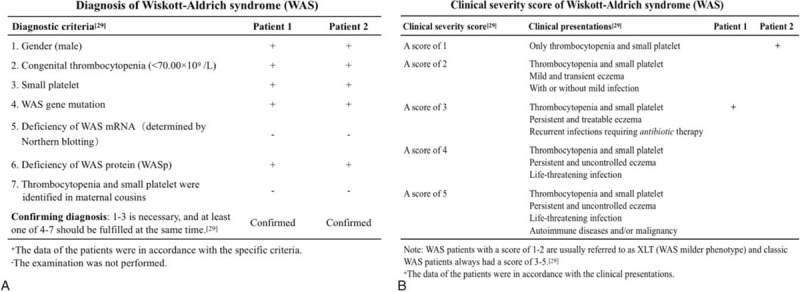
Diagnosis and clinical severity score of WAS. (A) Diagnosis of these 2 patients. Both patients fulfilled the necessary diagnostic criteria for WAS. (B) Clinical severity score of WAS. Patient 1 and Patient 2 were assigned a score of 3 (classic WAS) and a score of 1 (XLT), respectively. WAS = Wiskott–Aldrich syndrome, XLT = X-linked thrombocytopenia.

**Figure 9:**
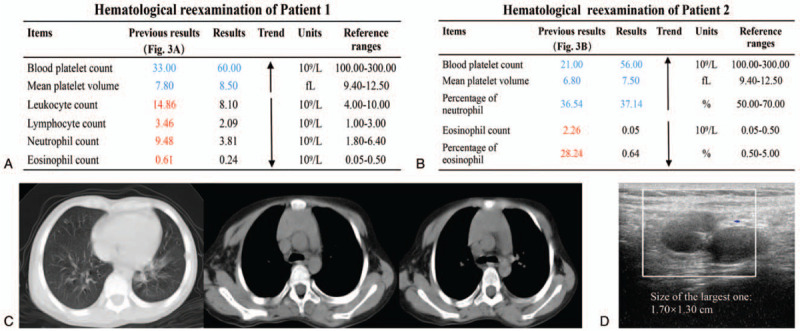
Hematological reexaminations of both patients and imaging reexamination findings of Patient 2. (A) Hematological reexaminations of Patient 1. Compared to the initial examinations (Fig. 3A), the counts of leukocyte, lymphocyte, neutrophil, and eosinophil returned to normal, and the platelet count and mean platelet volume (MPV) showed some recovery. (B) Hematological reexaminations of Patient 2. Compared to the initial examinations (Fig. 3B), the platelet count, MPV, and neutrophil percentage were partially recovered, and the count and percentage of eosinophil returned to normal. In (A) and (B), parameters above and below the reference range were depicted in red and blue, respectively. The upward (downward) arrow indicated an increase (decrease) in the parameter compared to previous results. (C) Image presentation of chest CT of Patient 2. No obvious mediastinal or hilar lymphadenopathy was found, and inflammatory lesions in bilateral lungs were significantly improved than before (Fig. 4F). (D) Abdominal color ultrasound of Patient 2. Multiple mesenteric lymph nodes were detected in the right lower abdomen and around the umbilicus, with the largest one of approximately 1.70 × 1.30 cm, which was significantly smaller than the size at admission (2.30 × 2.00 cm) (Fig. 4G). The white box indicates lymph nodes. CT = computed tomography.

Patient 1 showed a triad of thrombocytopenia-small platelets, eczema, and recurrent infections, while Patient 2 only experienced thrombocytopenia and small platelets without a previous history of recurrent infections. Combined with the obtained clinical data, the results of genetic and protein tests, and the diagnostic criteria published by the Pan-American Group for Immunodeficiency and European Society of Immunodeficiency in 1999 as well as a 5-point clinical severity scoring system of WAS,^[29]^ both Patient 1 and Patient 2 were diagnosed with WAS (Fig. 8A), and were classified as classic WAS (Patient 1, a score of 3) and XLT (Patient 2, a score of 1), respectively (Fig. 8B). In addition, Patient 2 was also confirmed to have pulmonary and abdominal tuberculosis based on the results of laboratory and imaging examinations. For Patient 1, desonide cream was used for eczema, traditional antibiotic (cefoperazone-sulbactam) was used for anti-infection, and immunoglobulins and platelets were intravenously transfused for supportive therapy. At present, the petechiae and eczema have disappeared, and vital signs were stable. Hematologic reexamination showed that, compared with abnormal results at admission (Fig. 3A), the counts of leukocytes (8.10 × 10^9^/L), lymphocytes (2.09 × 10^9^/L), neutrophils (3.81 × 10^9^/L), and eosinophils (0.24 × 10^9^/L) returned to normal, and the platelet count (60.00 × 10^9^/L) as well as MPV (8.50 fL) were partially recovered (Fig. 9A).

In addition to routine supportive treatment with intravenous immunoglobulins and platelet transfusions, Patient 2 who was diagnosed as XLT with abdominal and pulmonary tuberculosis received antituberculosis treatment with ethambutol and amoxicillin-clavulanate. Recently, the petechiae of his lower limbs have disappeared without any new bleeding spots. Hematological reexamination found that there was some recovery of the platelet count (56.00 × 10^9^/L), MPV (7.50 fL), and the neutrophil percentage (37.14%), but their levels were still below normative values. The eosinophil count (0.05 × 10^9^/L) and eosinophil percentage (0.64%) returned to normal (Fig. 9B). Furthermore, no obvious mediastinal or hilar lymphadenopathy was observed at the latest chest CT-scan, meanwhile, the inflammatory lesions in bilateral lungs (Fig. 9C) were significantly improved than before (Fig. 4F). Abdominal color Doppler ultrasound (Fig. 9D) revealed multiple mesenteric lymph nodes in the right lower abdomen and around the umbilicus, with the largest of about 1.70 × 1.30 cm, which was significantly smaller than that at admission (Fig. 4G). Regrettably, both patients discontinued the treatment for financial reasons.

## 4 Discussion

In this study, we reported for the first time a novel frameshift mutation (c.927delC, p.Q310Rfs∗135) on exon 9 of the *WAS* gene in Patient 1 (Fig. 5). Meanwhile, the XLT-related pathogenic mutation (c.1090C>T, p.R364X) in *WAS* gene has been reported, but this is the first time to verify that this mutation decreased the expression of WASp. The expression levels of WASp were examined by flow cytometry (Fig. 6), further demonstrating the pathogenicity of the 2 mutations. Exon 9 encodes the GBD domain of WASp, while exon 10 encodes the PRD domain and the Verprolin homology (V) of VCA domain (Fig. 1). The VCA domain is a key structure that interacts with the Arp2/3 complex and induces actin polymerization.^[20,21]^ In the inactive state, the VAC domain is hidden by a hairpin-like closed structure, when activated, the VCA domain is exposed and interacts with the Arp2/3 complex, thereby promoting the actin assembly.^[30,31]^ Meanwhile, many adaptor proteins containing Src homology 3 domains, such as Crk-Like protein and cell division cycle 42 interacting protein-4, interact with the PRD domain, relieve the WASp auto-inhibited conformation and release the VAC domain, thus phosphorylating and activating WASp.^[32,33]^ Therefore, mutations occurring on exons 9 and 10 of *WAS* gene may disrupt the structure and function of WASp by affecting corresponding coded domains.

The results of flow cytometry confirmed that the 2 mutations down-regulated the expression of WASp to varying degrees (Fig. 6). WASp expression was significantly decreased in PBMCs from Patient 1 with a severe phenotype (classic WAS) and relatively slightly decreased in PBMCs from Patient 2 with a milder phenotype (XLT). The difference of WASp expression in PBMCs from these 2 patients was consistent with the severity of their clinical phenotypes. Therefore, the results further elucidated that the severity of WAS is proportional to the degree of WASp deficiency. In addition, the analytical results of the lymphocyte subset also well reflected the immune status of both patients in this study (Fig. 7). In Patient 1 with obvious immunodeficiency, total T lymphocytes (CD3+: 49.58%) and CD8+ T lymphocytes (CD3+CD8+: 12.18%) were significantly decreased, while the lymphocyte subsets were normal in Patient 2 who had no apparent signs related to immunodeficiency. Combined with the low expression of WASp, the analytical results of lymphocyte subsets further demonstrated that significant WASp deletion could lead to T cell deficiency, which is a key factor in WAS-associated immune dysfunction. Among them, CD8+ T lymphocyte deficiency was the most common, and more than 50% of WAS patients were found to have reduced CD8+ T lymphocyte counts.^[11]^ In addition, Crowe et al proposed that a decreased number of CD4+ T lymphocytes could lead to an increased risk of opportunistic infection.^[34]^ Based on the analytical results of lymphocyte subsets for these 2 patients, we found that although their CD4+ T lymphocyte proportions were in the normal range, the proportion of CD4+ T lymphocytes in Patient 1 (CD3+CD4+: 30.87%) who had a history of repeated infection was lower than that in Patient 2 (CD3+CD4+: 40.36%). This phenomenon was consistent with the conclusion of Crowe et al.^[34]^ What's more, hematologic examination results of both patients on admission reflected varying degrees of eosinophil increase (Fig. 3). A previous study reported that eosinophilia was observed in 31% of WAS patients.^[3]^ Lexmond et al confirmed that this hematological characteristic resulted from the Tregs dysfunction caused by WASp deficiency, which weakened the suppressive function of Tregs on Th2 cell differentiation and consequently caused eosinophilia.^[35]^

We also identified a 3.10 mm diameter ASD in Patient 1 by UCG (Fig. 4A). ASD is the common birth defect in China. Typical hemodynamic change in the early stage of ASD is interatrial left-to-right shunt, which would increase blood flow in the pulmonary circulation.^[36]^ Pulmonary circulation can accommodate a large amount of blood. So, when the defect is small or at the early stage of the disease, normal pulmonary arterial pressure can still be maintained, without any obvious clinical symptoms or only with susceptibility to mild respiratory tract infections. If the defect is large and fails to be corrected in time, the long-term left-to-right shunt can gradually lead to pulmonary arteriolar intimal hyperplasia and increased medial thickness, as well as increased pulmonary vascular resistance, which may eventually progress into life-threatening pulmonary hypertension and right heart failure.^[37]^ Patient 1 with an ASD of merely 3.10 mm in diameter did not show obvious ASD-related symptoms (e.g., fatigue and exertional dyspnea). Due to the younger age (only 13 days old) of the patient and the small diameter (<8 mm) of the ASD, there is a high possibility of self-healing of the defect in the future, so the surgical repair of the defect is not currently performed. However, because of the immunodeficiency observed in Patient 1 with classic WAS, we will pay close attention to the clinical signs on him and regularly monitor the heart health by UCG to prevent pulmonary congestion or even acute heart failure induced by infection.

Based on imaging and laboratory examinations, Patient 2 was confirmed to be infected with tuberculosis. There have been only a few reports about classic WAS/XLT with tuberculosis.^[38,39]^ The environment is one of the decisive factors for the risk of tuberculosis infection in children,^[40]^ and tuberculosis in children is mostly caused by contact with adult patients with infectious tuberculosis, so we speculate that the tuberculosis infection of Patient 2 was derived from his father, who had active tuberculosis. Moreover, the risk of tuberculosis infection also depends on the immune function of the body. *Mycobacterium tuberculosis* (*Mtb*) belongs to intracellular parasitic bacteria, and cellular immunity mediated by macrophages and T lymphocytes plays a major role in the fight against *Mtb* infection.^[41,42]^ In this report, Patient 2 was classified as XLT, a relatively mild phenotype in patients with pathogenic *WAS* gene mutations. Although no prominent signs of immunodeficiency were shown in him, the relatively slight reduction of WASp might still have potential impacts on immune function, leading to increased susceptibility to *Mtb* and eventual progression to pulmonary tuberculosis as well as extrapulmonary tuberculosis. In addition, previous studies have confirmed that rifampicin and isoniazid may cause drug-induced thrombocytopenia during tuberculosis treatment.^[43,44]^ With the ability to act as haptens, both drugs above stimulate the body to produce antibodies and subsequently form antigen-antibody complexes, which are bound to platelets through Major histocompatibility complex class I antigens, resulting in the activation of complements, and eventually destroying platelets and increasing the risk of bleeding.^[45]^ Goto et al have reported that reduced platelet count is strongly associated with the death of tuberculosis patients during the treatment.^[46]^ Therefore, XLT patients with tuberculosis are not only easily misdiagnosed as drug-induced thrombocytopenia but also difficult to treat. Irrational use of antituberculosis drugs may aggravate thrombocytopenia and further increase the risk of death. During the course of Patient 2's treatment, we did not adopt the commonly used quadruple therapy of isoniazid, rifampicin, ethambutol, and pyrazinamide, but switched to ethambutol supplemented with amoxicillin-clavulanate for anti-tuberculosis treatment, which achieved significant effects (Fig. 9C and D). Noteworthily, for such young XLT patients, we still need to be vigilant to the possibility of XLT progressing to classic WAS with age, so long-term follow-up is strongly recommended.

Both of our patients received supportive therapy with intravenous immunoglobulins and platelet transfusions, but they abandoned further treatments for financial reasons. So far, allogeneic hematopoietic stem cell transplantation is the only mature radical treatment for WAS recognized worldwide, and the survival rate of WAS patients who received allogeneic hematopoietic stem cell transplantation ranges from 84% to 89.1%.^[47]^ In addition, gene therapy for WAS has attracted increased attention as a new therapeutic strategy, which can theoretically avoid transplant rejection and graft versus host disease.^[48]^ However, it is important to note that gene therapy is still in clinical trials and more studies will be needed to improve its safety.

In conclusion, WAS is a rare X-linked disorder that occurs in males. For male patients who present with congenital thrombocytopenia and small platelets, it is necessary to assess *WAS* gene mutation and WASp expression by the molecular genetic approach and protein detection as early as possible. In this study, we identified a novel pathogenic *WAS* gene mutation (c.927delC, p.Q310Rfs∗135) in a patient of Lahu (Patient 1). Researching and reporting novel pathogenic *WAS* gene mutations from different populations will expand the mutation spectrum of *WAS* gene and facilitate the genetic counseling and prenatal diagnosis. An ASD was also found in Patient 1, which was not reported in previous WAS-related researches. Is there a potential relationship between this novel mutation (c.927delC, p.Q310Rfs∗135) and heart development? This issue deserves further study. What's more, we also reported a rare case (Patient 2) of XLT with tuberculosis and described the clinical and molecular characteristics of the patient and the therapeutic regimen for him. However, the patient failed to complete the whole course of treatment, so the optimal combination therapy for classic WAS/ XLT patients with tuberculosis remains to be explored. We also examined the changes of WASp expression caused by *WAS* c.927delC/ c.1090C>T mutation to demonstrate that the severity of WAS was positively correlated to the degree of WASp deficiency. In addition, both of our patients in this study are Chinese ethnic minorities (Lahu and Zhuang). This might suggest that the distinct customs and environmental factors of ethnic minorities may lead to a high incidence of WAS. The genomic variations between ethnic minorities and other populations can be explored by genome-wide association analysis in future research.

## Acknowledgments

The authors sincerely thank the patients and their family members for their participation and support.

## Author contributions

**Data curation:** Haifeng Liu, Yanchun Wang, Lvyan Tao.

**Formal analysis:** Haifeng Liu, Yanchun Wang, Lvyan Tao, Yu Zhang.

**Funding acquisition:** Li Li, Yu zhang.

**Investigation:** Haifeng Liu, Yanchun Wang, Xiaoli He.

**Methodology:** Haifeng Liu, Yuantao Zhou, Xiaoning Liu, Yan Wang.

**Project administration:** Yangfang Li, Li Li.

**Writing – original draft:** Haifeng Liu, Yanchun Wang, Lvyan Tao.

**Writing – review & editing:** Yangfang Li, Li Li.
